# Absorption of Hazardous Pollutants by a Medicinal Fern *Blechnum orientale* L.

**DOI:** 10.1155/2013/192986

**Published:** 2013-07-15

**Authors:** Xiao-min Zhu, Yuan-wen Kuang, Dan Xi, Jiong Li, Fa-guo Wang

**Affiliations:** ^1^Key Laboratory of Vegetation Restoration and Management of Degraded Ecosystems, South China Botanical Garden, Chinese Academy of Sciences, Guangzhou 510650, China; ^2^University of Chinese Academy of Sciences, Beijing 100049, China; ^3^Key Laboratory of Plant Resources Conservation and Sustainable Utilization, South China Botanical Garden, Chinese Academy of Sciences, Guangzhou 510650, China

## Abstract

A Chinese medicinal fern *Blechnum orientale *(Linn) was separately collected from polluted and unpolluted sites to determine whether it could accumulate hazardous pollutants or not. Metal concentrations (Cu, Zn, Mn, Pb, Cd, Cr, As, and Hg) both in the fronds and roots and polycyclic aromatic hydrocarbons (PAHs) in the fronds of this fern were quantified. At both sites, roots of *B. orientale *had significantly higher heavy metals than the fronds. Concentrations of Pb, As, Hg, Cd, and Cu in the fronds at the polluted site were more than 2, 6, 7, 14, 5, and 13 times of those at the unpolluted site. Translocation factor and bioaccumulation factor implied that *B. orientale *did not have a good ability to transport heavy metals from the roots to the fronds. Total PAHs in the fronds at the polluted site were significantly higher than those at the unpolluted site, indicating the physiological PAHs absorption by *B. orientale *growing at polluted sites. Uptake of pollutants via stomata might be the main reason causing the significant accumulation of hazardous pollutants in the fronds of *B. orientale*. Large-scale systematical survey and intensive monitoring on pollutants in this medicinal fern should be necessarily strengthened.

## 1. Introduction

Various plants have been identified as traditional medicines for pharmaceutical and dietary therapy by folks for several millennia. Eighty percent of the world's inhabitants were estimated to rely mainly on their traditional medicines for health care [1]. Recently, the search for phytoconstituents possessing antioxidant, anticancer, and antimicrobial properties from medicinal plants has been globally escalated [2]. With the rapid development and wide employment of modern analytical equipment and technology, more and more antioxidants have been isolated from different plant materials [3, 4]. It is estimated that up to 1994 approximately two-thirds of anticancer drugs approved worldwide were derived from plant sources [5].

**Figure 1:**
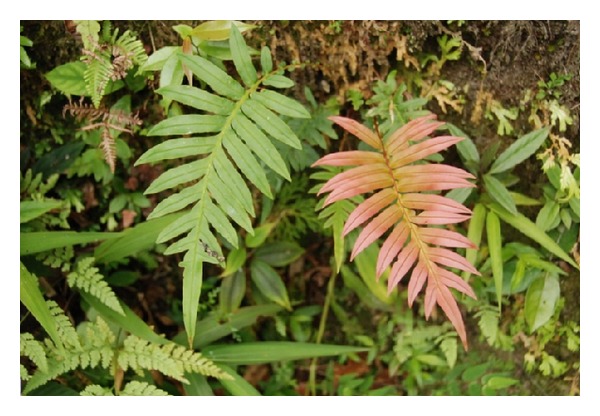
Photo of *Blechnum orientale *L. (*Kuan Chung* in Chinese). The species was authenticated by Dr. Fa-guo Wang from Key Laboratory of Plant Resources Conservation and Sustainable Utilization, South China Botanical Garden, Chinese Academy of Sciences, Guangzhou, China. The species for this study was sampled in August 2010. The voucher specimen (voucher no. 01721757) has been kept in Chinese Virtual Herbarium, Chinese Academy of Sciences.

*Blechnum orientale *L. (*Kuan Chung* in Chinese, Figure 1) has been used as ethnomedicine since the ancient time [6, 7]. Due to the ongoing search for natural antibacterial and antioxidant agents from medicinal ferns, researchers have validated the potential of  *B. orientale* as broad-spectrum bactericide [8], antioxidant agent for cancer therapy [3], antimicrobial activity [8], and candidate for the treatment of dermal wounds [9]. Presently, there have been increasing interests in phytochemistry of this medicinal fern, however, there has been relatively less consideration on the risk of hazardous pollutants in tissues of *B. orientale*. In a field investigation, *B. orientale* was found to grow vigorously even with extremely high levels of arsenic (As, 142 mg·kg^−1^) in the fronds at sites severely polluted by heavy metals [10]. Usually, the concentration of As was below 1.0 mg·kg^−1^ in most terrestrial plants [11].

 The higher levels of toxic heavy metals (i.e., As, Pb) than their limited concentrations in “China Green Trade Standards of Importing and Exporting Medicinal Plants and Preparation (GTSIEMPP)” in some Chinese traditional medicines might press potential risk to human health. Historically, many ferns have been consumed as edible plant and utilized as pharmaceutical species in China. The accumulation and/or the adsorption of toxic pollutants by medicinal ferns have not been intensively concerned up to date. The main objectives of this study were to (1) detect the degree to which *B. orientale* accumulated toxic metals and polycyclic aromatic hydrocarbons (PAHs) in a polluted environment and (2) investigate the transportation of heavy metals from roots to fronds of *B. orientale*.

## 2. Materials and Methods

### 2.1. Site Description and Sample Collection

Specimens of *B. orientale* were collected from two woodlands with different levels of pollution. The first woodland located about 5 km away from a Zn-Pb smelting plant. The second woodland located in a natural reserve. Previous investigation showed that the woodland nearby the smeltery was a polluted site (PS), and the woodland in the reserve was a relatively unpolluted site (UPS) [12]. At each woodland, the fronds and roots were randomly and separately collected from ten clusters of *B. orientale*. The clusters were at least 100 m apart and were at least 200 m from the nearest road. About 500 g of fronds and 500 g of roots (fresh weight) were collected from each cluster. Surface soils (0–10 cm depth) beneath each cluster were sampled at the same time. The fronds, roots, and soils from each cluster were treated as a replicate. Thus, ten replicates of fronds, roots, and soils were collected at each site. All samples were kept in paper envelopes and then sealed in polythene bags before being transported to the laboratory.

### 2.2. Laboratory Analyses

In the laboratory, the fronds were quickly cleaned with water and then rinsed with deionized water. The roots were first cleaned with water and then rinsed with an ultrasonic cleaning instrument to remove remaining soil particles. All samples were then dried at 65°C to constant weight, ground, and stored in plastic vials for heavy metals analyses. A portion of each frond sample (fresh) was gently washed with tap water, rinsed with deionized water, freeze-dried for 36 h, and ground for PAHs detection.

Soil samples were air-dried at room temperature, lightly crushed, and passed through 2 mm acid-washed plastic sieve to remove stones, roots, leaves, and other debris. All samples were ground in a mortar, passed through a 100-mesh polyethylene sieve, and were kept in desiccators.

 Concentrations of heavy metals (Cu, Zn, Mn, Pb, Cr, Cd, As, and Hg) and PAHs were determined separately. Samples for metal analyses were digested with concentrated HNO_3_ in a microwave digestion system. Metal concentrations were quantified with an inductively coupled plasma-mass spectrometer (ICP-MS, Agilent 7500CX, USA). Precision and accuracy of the determinations were tested through blank and repeated analyses of National Standard Reference material (GBW 07604).

PAHs in fronds were extracted and quantified according to the method detailed by Sun et al. [13]. The 16 EPA priority PAHs were analyzed by GC-MS (Thermo Trace DSQ, USA) in selected ion monitoring (SIM) mode. Total PAHs (∑PAHs) were defined as the sum of the 16 EPA priority PAHs. 

### 2.3. Statistical Analyses

The results for the concentrations of heavy metals and PAHs were presented as mean and standard deviation (SD). Paired sample *t*-tests were used to compare the differences of pollutant concentrations in the fronds, roots, and soils between the sites at the confidence level of 95% using software SPSS 10.0 (SPSS Inc., Chicago, IL, USA).

## 3. Results

### 3.1. Comparison of Metals in Soils

Concentrations of the selected heavy metals beneath the ferns at both sites were compared in Table 1. As demonstrated, concentrations of all the selected heavy metals in the soils at the polluted site were significantly higher than those at the unpolluted site. The comparison indicated that the woodland nearby the smelting plant has received substantial atmospheric metal deposition probably originating from the Pb-Zn smeltery.

### 3.2. Heavy Metals Absorbed by the Fern

**Figure 2:**
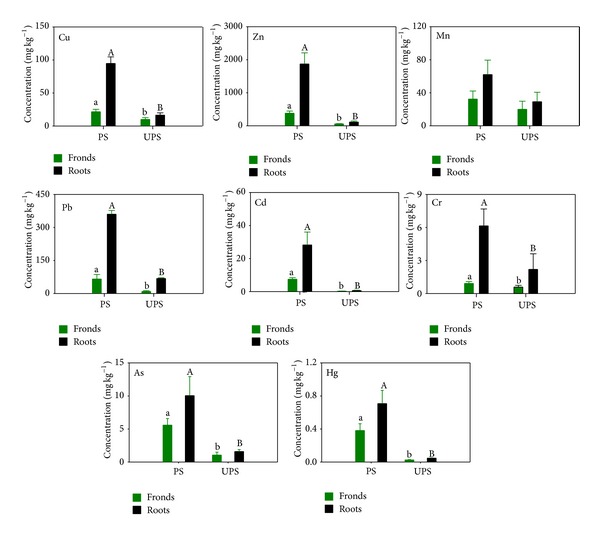
Concentrations (means ± SD, mg·kg^−1^ DW, *n* = 10) of heavy metals in the fronds and the roots of *B. orientale* sampled at the woodland nearby a smeltery (polluted site, PS) and in a natural reserve (relatively unpolluted site, UPS). For each plant part in each panel, bars with different letters indicate significant differences (*P* < 0.05) between the sites.

Concentrations of the selected heavy metals in the fronds and roots of *B. orientale* at the two sites were shown in Figure 2. At both sites, the order of the heavy metals in terms of concentration in both the fronds and the roots was Zn > Pb > Cu > Mn > Cd > As > Cr > Hg. Zinc, Pb, and Cu were the most abundant, while Hg, Cr, and As were the least metals in the fern.

At both sites, the roots of *B. orientale* always had significantly higher metal concentrations than the fronds. All the studied heavy metals except for Mn both in fronds and roots from the polluted site were significantly higher than those from the unpolluted site. For instance, fronds Cu, Zn, Pb, Cd, As, and Hg at the polluted site were more than 2, 6, 7, 14, 5, and 13 times of those at the unpolluted site, respectively. Concentrations of Mn both in fronds and roots were similar between the sites.

### 3.3. PAHs in the Fronds

Concentrations of total PAHs (∑PAHs) and individual PAHs in the fronds of *B. orientale* were presented in Table 2. The ∑PAHs and 10 of the 16 PAHs in the fronds at the polluted site were significantly higher than those at the unpolluted one. Concentrations of the other 6 PAHs in fronds did not differ between the sites.

**Figure 3:**
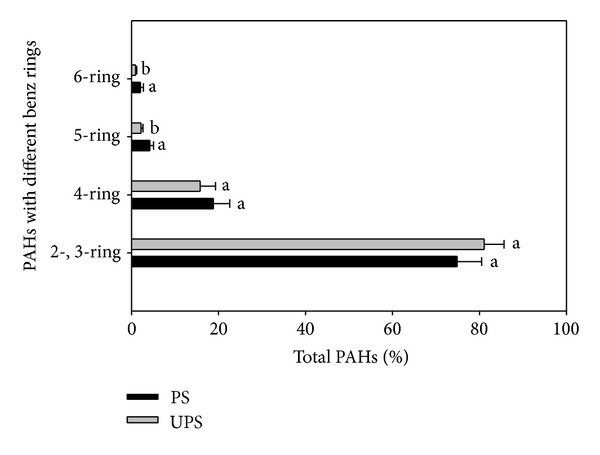
Percentages (mean ± SD) of 2-, 3-, 4-, 5-, and 6-ringed PAHs in the fronds of *B. orientale* sampled at the polluted site (PS) and the relatively unpolluted site (UPS). Significant differences were marked with different letters at the levels of *P* < 0.05.

For further analysis of the PAH composition in the fronds, we grouped the PAH compounds into classes according to the number of benzene rings in their structures (Figure 3). Low molecular-weight PAHs (2- and 3-ringed compounds, LMW-PAHs), especially Nap, dominated and accounted for more than 70% of ∑PAHs in the fronds, while 4-ringed PAHs equaled at both sites. The fraction of LMW-PAHs at the polluted site was lower than that at the unpolluted site, but the fraction of higher molecular-weight PAHs (HMW-PAHs), in particular 5- and 6-ringed compounds, at the polluted site was higher than that at the unpolluted site.

## 4. Discussion

The selected heavy metals in the soils at the polluted site were extremely above their background values in Guangdong forest soils [14]. The results coincided well with the differences in atmospheric deposition of heavy metals between the two sites [12]. Recent study confirmed that concentrations of PAHs in plant tissues close to the smeltery were significantly higher than those at rural site [13]. Therefore, in this study, we considered the woodland nearby the smeltery to be a severely polluted site and the one in the natural reserve to be a relatively unpolluted site.

Heavy metals accumulated in medicinal plants could pose potential toxicity for human being due to the biomagnified effect along food chains [15]. Many fern species have been identified to absorb metals through the roots [16] and to accumulate toxic metals (i.e., As) in the fronds [17]. According to the heavy metal limitation for Chinese traditional medicines (GTSIEMPP), total concentrations of Pb, As, Hg, Cd, and Cu should be lower than 20.0, 5.0, 2.0, 3.0, 0.3, and 20.0 mg·kg^−1^, respectively, in raw materials of medicinal plants. In this study, the average values for Pb, As, Hg, Cd, and Cu in the fronds of *B. orientale* nearby the smelting plant were 66.0, 5.6, 0.4, 7.5, and 21.7 mg·kg^−1^, respectively, which indicated that Pb, As, Cd, and Cu were above the standard of GTSIEMPP. In contrast, the concentrations of Pb, As, Hg, Cd, and Cu in the fronds at the natural reserve were within the limitation of GTSIEMPP. The results implied potential risk on human health when this fern was collected for medicinal and edible utilization from polluted environments.

To estimate the metal transportation from roots to fronds and the metal bioaccumulation from soil to fronds, we compared the translocation factor (TF) and bioaccumulation factor (BF), which was the ratio of metal concentration in aboveground tissues to that in roots [15] and the ratio of concentration in soils to that in aboveground tissues [18], respectively, of this fern. Notably, the fern of *B. orientale* had both TF and BF values less than 1.0 at both sites (Table 1, Figure 2). A TF value < 1 indicated the immobilization of metals in the roots [15], and a BF < 1 showed the exclusion of metals from soils [18]. Based on the factors, we inferred that *B. orientale* did not have a good ability to transport heavy metals from the roots to the fronds. The metals in the fronds of *B. orientale *might be the atmosphere absorption via stomata rather than the soil uptake through roots. This consequence was in agreement with that reported by Oliva and Espinosa [19]; namely, atmospheric deposition is one of the major pathways by which metals entered plant leaves. Additionally, roots *B. orientale* might have acted as a storage organ of the metals after the uptake from atmosphere. This was especially true for most of the metals since higher concentrations of metals were recorded in the roots and lower concentrations in the fronds. The accumulation of metals in the roots rather than in the fronds could partly reduce the potential toxicity to human being when the tender fronds were collected for edibles. 

Polycyclic aromatic hydrocarbons were another common pollutant with carcinogenic, mutagenic, and teratogenic effects on organisms. Environmental PAHs were mainly from multiply anthropogenic emissions. After being emitted into the atmosphere, PAHs could commonly be absorbed in a vaporous or particulate state by the aboveground tissues of plants. The concentrations of most PAHs in plant tissues were determined by the atmospheric deposition of PAHs from the atmosphere. In the present study, the significantly higher concentrations of both ∑PAHs and several individual PAHs in the fronds from the polluted site indicated that *B. orientale* could accumulate PAHs as well as heavy metals in fronds. The accumulation of PAHs suggested that consumption and utilization of *B. orientale* fronds growing at polluted sites could be detrimental to human health. 

The ratios of LMW-PAHs to HMW-PAHs were often used to discriminate the origins (petrogenic versus pyrogenic) of airborne PAHs [20]. In this study, LMW-PAHs represented more than 70% of the PAHs in the fronds at both sites, suggesting a pyrogenic origin of PAHs in the fronds. Since LMW-PAHs were chiefly generated by low- or moderate-temperature combustion process (such as biomass burning and domestic coal burning) while HMW-PAHs were mainly generated by high-temperature combustion process (such as vehicular exhaust and industrial coal combustion), the larger fraction of LMW-PAHs in the fronds from both sites indicated that PAHs in the fronds of  *B. orientale* might mainly stem from low- or moderate-temperature combustion processes. The higher fraction of HMW-PAHs in the fronds implied that there were additional industrial sources at the polluted site. Clarifying the contributions of these different sources required further quantification of PAHs in the fern, soil, and atmosphere at the both sites. 

## 5. Conclusion

The results of this case study revealed the significant adsorption and accumulation of toxic metals and hazardous PAHs by* B. orientale* growing at polluted site and confirmed the nontransportation of heavy metals from roots to fronds. The substantial higher concentrations of the studied heavy metals and PAHs implied potential risk of toxicity on human health when this medicinal fern was harvested from polluted sites. Large-scale systematical survey and intensive monitoring on pollutants in medicinal ferns should be necessarily strengthened. The collection of *B. orientale* for medicinal utilization from polluted areas should better be abandoned to reduce the intake of hazardous pollutants by human being.

## Figures and Tables

**Table 1:** Concentrations of heavy metals in the surface soils (mean ± SD, mg·kg^−1^ DW, *n* = 10) at the polluted site and unpolluted site. Background values for the selected heavy metals in the forest soils of Guangdong province were also presented.

Metal	Polluted site	Unpolluted site	Background value^b^
Cu	80.75 ± 3.03^∗a^	3.69 ± 0.72	9.63 ± 2.66
Zn	721.45 ± 18.26*	19.58 ± 4.01	32.52 ± 1.83
Mn	1017.26 ± 68.65*	44.82 ± 11.20	110.89 ± 2.18
Pb	965.58 ± 252.57*	33.76 ± 9.32	24.06 ± 1.96
Cr	928.63 ± 103.19*	39.64 ± 9.27	39.08 ± 2.07
Cd	7.17 ± 1.46*	0.085 ± 0.014	0.03 ± 2.54
As	21.89 ± 1.48*	8.62 ± 0.55	9.31 ± 2.47
Hg	1.25 ± 0.12*	0.10 ± 0.06	0.051 ± 1.76

^a^An asterisk (∗) indicates a significant difference (*P* < 0.01) between the two sites according to a paired sample *t*-test; ^b^background values in forest soils in Guangdong province [13].

**Table 2:** Concentrations (mean ± SD, ng·g^−1^ DW, *n* = 10) of PAHs detected in the fronds of *B.  orientale* at the polluted site and the relatively unpolluted site.

PAH	Polluted site	Unpolluted site
Nap	87.27 ± 19.12*	73.54 ± 20.67
Acpy	0.52 ± 0.18	0.83 ± 0.63
Acp	0.65 ± 0.28	0.60 ± 0.34
Flu	22.19 ± 4.39	32.60 ± 14.63
PA	99.18 ± 10.40*	56.81 ± 26.77
Ant	7.68 ± 2.30*	2.14 ± 0.72
FL	6.42 ± 1.69	4.80 ± 1.85
Pyr	16.66 ± 2.81	11.68 ± 3.34
BaA	8.44 ± 3.23*	2.98 ± 1.18
Chr	9.59 ± 2.59	12.77 ± 3.95
BbF	3.03 ± 1.22*	0.89 ± 0.34
BkF	16.61 ± 4.75*	4.02 ± 1.99
BaP	2.19 ± 0.61*	1.43 ± 0.46
DBA	4.11 ± 2.13*	1.10 ± 0.58
IND	6.11 ± 2.58*	1.93 ± 1.25
BghiP	6.05 ± 2.15*	1.96 ± 0.42

∑PAHs	290.68 ± 22.73*	210.09 ± 36.07

An asterisk (∗) indicates a significant difference (*P* < 0.05) between the two sites according to a paired sample *t*-test.
